# Hydroxy- and Amino-Phosphonates and -Bisphosphonates: Synthetic Methods and Their Biological Applications

**DOI:** 10.3389/fchem.2022.890696

**Published:** 2022-06-01

**Authors:** Babak Kaboudin, Payam Daliri, Samaneh Faghih, Hesam Esfandiari

**Affiliations:** Department of Chemistry, Institute for Advanced Studies in Basic Sciences, Zanjan, Iran

**Keywords:** phosphonates, bisphosphonates, inhibitors, medicine, insecticides, osteoporosis

## Abstract

Phosphonates and bisphosphonates are stable analogs of phosphates and pyrophosphates that are characterized by one and two carbon–phosphorus bonds, respectively. Among the various phosphonates and bisphosphonates, hydroxy and amino substitutes are of interest as effective in medicinal and industrial chemistry. For example, hydroxy bisphosphonates have proven to be effective for the prevention of bone loss, especially in osteoporotic disease. On the other hand, different substitutions on the carbon atom connected to phosphorus have led to the synthesis of many different hydroxy- and amino-phosphonates and -bisphosphonates, each with its distinct physical, chemical, biological, therapeutic, and toxicological characteristics. Dialkyl or aryl esters of phosphonate and bisphosphonate compounds undergo the hydrolysis process readily and gave valuable materials with wide applications in pharmaceutical and agriculture. This review aims to demonstrate the ongoing preparation of various classes of hydroxy- and amino-phosphonates and -bisphosphonates. Furthermore, the current review summarizes and comprehensively describes articles on the biological applications of hydroxyl- and amino-phosphonates and -bisphosphonates from 2015 until today.

## Introduction

In living organisms, the phosphorus atom is one of the main elements that have an important role in biochemical reactions (Krazewski et al., 2020). Among the wide range of phosphorus compounds, phosphates and pyrophosphates have important roles in living organisms (Elliott et al., 2012). Phosphonates and bisphosphonates are stable analogs of phosphates and pyrophosphates that represent an important class of bioisosteres for chemical biologists and medicinal chemists (Park et al., 2021) (Horsman and Zechel 2017). The replacement of the P-O bond with the P-C bond increases their chemical and enzymatic stability. One of the highlighted applications of phosphonates has been reported in the COVID-19 pandemic (Jockusch et al., 2020). Among the various phosphonates and bisphosphonates, hydroxy- and amino-phosphonates and -bisphosphonates are of interest as effective in medicinal and industrial chemistry (Ebetino et al., 2022) (Santos et al., 2020). For example, hydroxy bisphosphonates are well-known bone disease drug candidates, and there are eight clinical hydroxy bisphosphonate drugs that have been introduced for bone diseases due to their high tendency to bind hydroxyapatite, the bone mineral compounds.

Hydroxy- and amino-phosphonates and -bisphosphonates have been prepared by various methods. First in this review, a short description will be presented ongoing the preparation methods of hydroxy- and amino-phosphonates and -bisphosphonates. Furthermore, the biological application of hydroxyl- and amino-phosphonates and -bisphosphonates from 2015 until today will be summarized and comprehensively described.

## Synthetic Methods for the Preparation of Hydroxy- and Amino-Phosphonates and-Bisphosphonates

### Hydroxyphosphonates

Hydroxyphosphonates and phosphonic acids inhibit enzymes such as renin, EPSP synthase HIV protease, and PTPases. Other biologically significant *α*-substituted phosphonates and phosphonic acids are also readily obtainable from hydroxyphosphonates (**1**). The base-catalyzed hydrophosphonylation of aldehydes (the Pudovik reaction) is one of the most important methods for the synthesis of hydroxyphosphonates (Sardarian and Kaboudin, 1997) (Supplementary Scheme S1 in SI).

In another method, the synthesis of hydroxyphosphonates has been reported from the reaction of carbonyl compounds with the addition of nucleophilic trialkyl phosphate (Vasikaran, 2001). In general, both reactions are performed in the presence of a catalyst and in some cases in the presence of the base (Radai 2019) (Radai and Keglevich 2018b). In recent years, enantiomerically pure hydroxyphosphonates have been synthesized by chiral-resolving agents or asymmetric chiral synthesis (Rádai et al., 2018a; Kaboudin et al., 2019a).

### Aminophosphonates

Aminophosphonates **2** is the most common category of phosphonate esters. A number of synthetic methods for the synthesis of aminophosphonates have been developed during the past two decades (Amira et al., 2021). Of these methods, the Kabachnik–Fields (Kaboudin and Moradi, 2005) (Bhagat and Chakraborti, 2007) synthesis of 1-aminophosphonates, catalyzed by a base or an acid, is the most convenient method (Varga and Keglevich 2021) (Sravya et al., 2021) (Supplementary Scheme S2 in SI).

The Kabachnik–Fields synthesis of aminoalkyl phosphonates is the nucleophilic addition of an amine to a carbonyl compound followed by the addition of a dialkyl phosphite to the resulting imine. Lewis acids such as InCl_3_, SnCl_4_, BF_3_, Et_2_O, ZnCl_2_, and MgBr_2_, have been used as catalysts. The aminophosphonates have also been synthesized by various other methods such as 1)) addition of P-H function to nitriles (Gancarz and Wieczorek, 1978), 2)) Arbuzov and Michaelis–Becker reactions (Seyferth et al., 1971), 3)) reaction of hydroxyphosphonate with amines (Kaboudin, 2003), 4)) condensation of X-NH_2_ with acyl phosphorus species (Worms et al., 1976), 5)) Curtius and Hofmann rearrangement of substituted phosphonoacetic esters (Barycki et al., 1970), and 6)) alkylation of nucleophilic precursors such as Schiff bases (Chandrasekhar et al., 2001). It seems that the Kabachnik–Fields reaction is the most efficient one among these routes. This reaction can be catalyzed by other catalysts such as BiCl_3_ (Zhan and Li, 2005), SnCl_2_ (Gallardo-Macias and Nakayama, 2010), CaCl_2_ (Kaboudin and Zahedi, 2008), or/and PPh_3_ (Tian et al., 2009). The reaction also can be promoted by heating or microwave irradiation (Kaboudin and Nazari, 2001; Ranu and Hajra, 2002; Macarie et al., 2019). In recent years, asymmetric synthesis of aminophosphonates has also been reported (Maestro et al., 2018, 2019; Maestro et al., 2020; Maestro et al., 2021).

### Hydroxybisphosphonates

The hydroxybisphosphonic acid derivatives **3** are important groups within organophosphorus pharmaceutics. Due to the high complexation ability of these compounds with calcium ions, the resorption of these ions is prevented. These compounds are used in the treatment of Paget’s disease, osteoporosis, and hypercalcemia. One of the most widely used methods for the synthesis of hydroxybisphosphonates is the reaction of carboxylic acids with phosphorus trichloride and phosphoric acids and followed by hydrolysis with water (Kieczykowski et al., 1995) (Supplementary Scheme S3 in SI).

The reaction proceeded *via* the formation of an ketophosphonate intermediate by the cross-linking of phosphorus nucleophile with the formed acyl chloride. In another process, the 1-hydroxy-1,1-bisphosphonates were prepared in high yield by the phosphite addition to ketophosphonates prepared from the Arbuzov reaction (Lecouvey and Leroux, 2000).

### Aminobisphosphonates

Aminobisphosphonates are important bisphosphonates with strong inhibitors of bone resorption that several typical structures of these compounds have been commercialized as drugs for the treatment of osteoporosis, Paget’s disease, and fibrous dysplasia (Chmielewska and Kafarski, 2016a) (Chmielewska and Kafarski, 2016b). Furthermore, due to their ability to complex metal ions, aminobisphosphonates have found important industrial applications, mainly, as corrosion inhibitors. Due to increasing interests in the biological activity of aminobisphosphonates, the development of methods for their synthesis is growing. Although several general procedures were previously elaborated to reach this goal, aminobisphosphonate chemistry is still developing quite substantially. Additionally, selected examples of aminobisphosphonate derivatization illustrate their usefulness for obtaining new diagnostic and therapeutic agents. A number of synthetic methods for the synthesis of 1-aminobisphosphonates have been developed using various starting materials (Kaboudin et al., 2019b) (Supplementary Scheme S4 in SI). One of the most important methods in the synthesis of 1-aminobisphosphonates is the use of amides or nitriles as substrates. Amides and nitriles are available in a wide variety of compounds and are easy to prepare.

However, there are many other procedures reported for the synthesis of aminobisphosphonates using isonitriles, oxophosphonates, and vinylidene bisphosphonates (Plöger et al., 1972; Fukuda et al., 1975; Bandurina et al., 1978; Wu et al., 2004; Szajnman et al., 2005; Yu et al., 2008; Roth et al., 2009; Midrier et al., 2011; Goldeman et al., 2012; Rodriguez, 2014). A wide range of aminomethylene bisphosphonic acids can be obtained from the simple three-component of amines, diethyl phosphite, and triethyl orthoformate (Kaboudin and Alipour, 2009).

## Biological Activation of Hydroxy- and Amino-Phosphonates and -Bisphosphonates

Hydroxy- and amino-phosphonates and -bisphosphonates are an important class of compounds that are currently receiving significant attention. Various hydroxy- and amino-phosphonates and bisphosphonates structures have been synthesized and described in the literature with interesting applications. The growing interest in the biological activity of these compounds has stimulated the development of their applications.

### Medicinal Applications

Treatment of *tuberculosis* (TB) is problematic due to the emergence of *Mycobacterium tuberculosis* (Mt), so a new drug is needed. One of these targets is hypoxanthine-guanine phosphoribosyltransferase (HGPRT), which synthesizes 6-oxopurine nucleoside monophosphates, which are essential for DNA/RNA production. Combination of [3R, 4R]-4-hypoxanthin-9-yl-3-((S)-2-hydroxy-2-phosphonoethyl) oxy-1-N-(phosphonopropionyl)pyrrolidine and [3R, 4R]-4-guanin- 9-yl-3-((S)-2-hydroxy-2-phosphonoethyl)oxy-1-N(phosphonopropionyl)pyrrolidine is the most potent inhibitor of MtHGPRT (compound **5**). This drug has low toxicity in mammalian cells (CC50 of 132 ± 20 μM). Therefore, it is a good inhibitor for anti-tuberculosis chemotherapy (Supplementary Scheme S5 in SI) (Eng et al., 2018).

Cheviet et al. (2020) reported the synthesis and biological activities of 24 novel hydroxyphosphonic acid derivatives, including 22 new non-cyclic nucleoside phosphonates. The compounds were studied as inhibitors of P. falciparum. Biological assays of phosphonic acid compounds (as sodium salts) on cell cultures revealed that the compounds’ effectiveness completely depends on the hydroxyl group, the chain length, and the nature of the base. Of all the compounds, derivative (R)-(4-(2-amino-6-oxo-9H-purin-9-yl)-2-hydroxybutyl)-phosphonic acid **6** appears to have the most suitable characteristics (i.e., guanine as nucleobase, a butyl chain, and a hydroxy group in the *β*-position and with R stereochemistry). It showed remarkable *in vitro* activity against P. falciparum-infected red blood cells (IC_50_ = 74 nM), and it has a high selectivity index (SI > 1,350). The compound has no toxicity on human cell lines. This was the first report on the antiplasmodial activity (*in vitro* and *in vivo*) of an acyclonucleoside phosphonate derivative (Supplementary Scheme S6 in SI).

**FIGURE 1:**
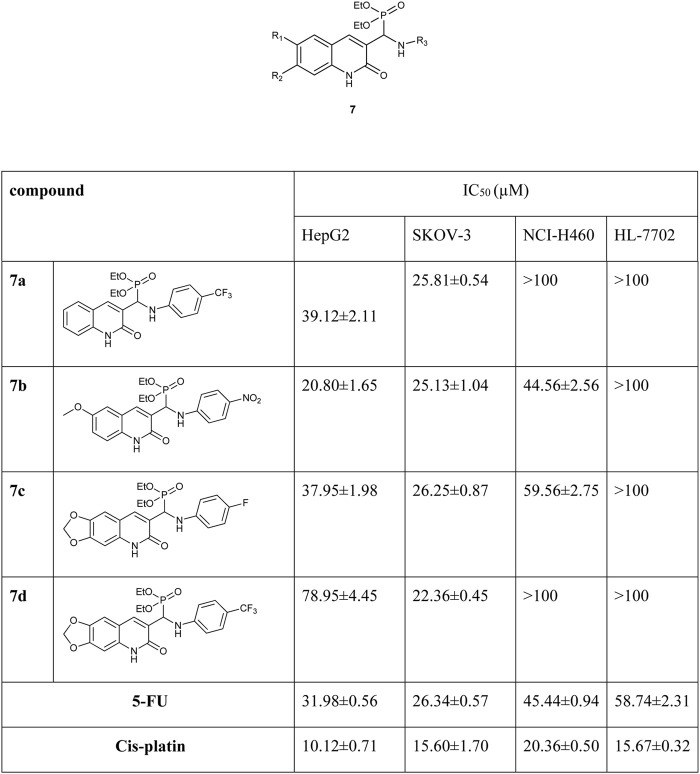
Structure of the compound **7** and its biological results.

The compounds **7** synthesized by the Kabachnik–Fields reaction. The *in vitro* cytotoxicity of the compounds was evaluated against HepG2 (human liver cancer cell line), SK-OV-3 (human ovarian cancer cell line), NCI-H460 (human large cell lung cancer cell line), and HL-7702 (human liver normal cell line) cell lines. The results show that, in HepG2 assay, compounds **7c** exhibit more cytotoxicity activity than 5-FU drug. In SK-OV-3 assay, **7a** and **7d** exhibit more cytotoxicity activity than 5-FU. In NCI-H460 assay, **7c** has more anticancer properties than 5-FU (Figure 1) (Yu et al., 2017).

Recently, a novel series of *α*-aminophosphonates **8** were synthesized through the Ugi three-component reaction (Supplementary Scheme S7 in SI). The compound **8** derivatives with R_1_: Bn, R_2_: Ph, and R_3_: Cy, and R_1_: Me, R_2_: *p*-SCCl_3_Ph, and R_3_: Cy showed good cytotoxicity against the A549 cell line (carcinomic human alveolar basal epithelial cell). They presented an IC_50_ Value of 16.14 ± 1.14 µM and 14.56 ± 2.53 µM, respectively (López-Francés et al., 2021). The same group also reported antiproliferative effect on A549 cells of some novel a-aminophosphonic acid derivatives **9–11** synthesized through the addition of O- and S- nucleophiles to 2H-azirines (Supplementary Scheme S8) (Carrraminana et al., 2020). All the compounds **9–11** showed selectivity on cancer cells (A549) over non-malignant cells (MCR-5).

Zhang et al. (2020) reported the synthesis and biological activities of novel sulfonamide-containing aminophosphonates **42** derivatives (Supplementary Scheme S8 in SI). Among the synthesized compounds 42, a derivative with R_1_ = 3-methoxy and R_2_ = methyl exhibited applicative COX-2 inhibitory (IC_50_ = 0.28 ± 0.05 µM). This compound also showed anticancer properties even more than cisplatin commercial drug. IC_50_ (µM) values of this aminophosphonate is 9.71 ± 0.47 for HeLa (human cervical cancer cells), 16.43 ± 0.62 for MCF-7 (human breast cancer cells), 2.34 ± 0.27 for HCT116 (human colon cancer cells), 12.51 ± 1.18 for HepG2 (human liver cancer cells), and 205.95 ± 2.36 for 293T (epithelial cell line).

**FIGURE 2:**
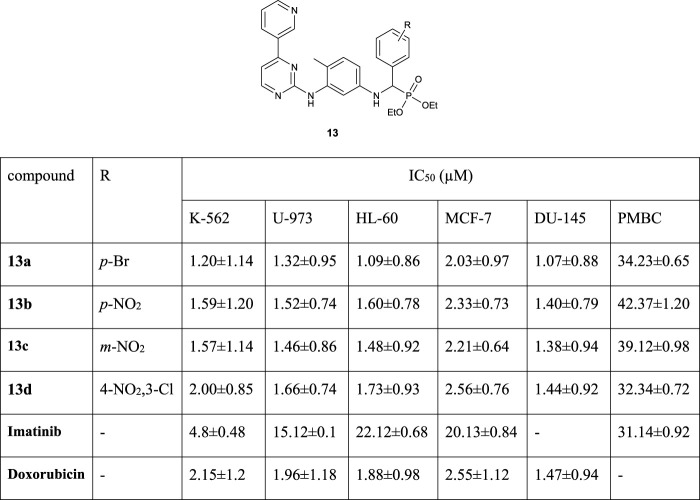
Structures of the compound **13** and its biological activities.

Aita et al. (2021) reported the synthesis and biological activities of aminophosphonate derivatives of imatinib. According to their results *in vitro* cytotoxicity assay against human leukemia cells (K-562, U-973, and HL-60), human breast cancer cell (MCF-7), and human prostate cancer cell (DU-145) for the compounds **13a–d** showed more anticancer activity than imatinib and doxorubicin drug (Figure 2).

One of the causes of Alzheimer’s disease is a lack of acetylcholine in the brain, so aminophosphonates as acetylcholine esterase (AChE) inhibitors can be used to prevent and treat Alzheimer’s. Shaikh et al. (2020) reported the synthesis and anticholine esterase activities of a series of novel N-substituted pyrazole-derived *α*-aminophosphonates **14**. All the synthesized aminophosphonates were tested for their inhabitation of acetylcholine esterase by Ellman’s method. Among them, compounds **14a** and **14b** showed better activities than standard drugs (tacrine, rivastigmine, and galantamine). The antioxidant activities of the synthesized aminophosphonates **14** were assayed (Table 1).

**TABLE 1:** Antichloline esterase activities of compound **14**.

Compound		AChE IC_50_ (µM)	DPPH method IC_50_ (µM)	H_2_O_2_ method IC_50_ (µM)
**14a**	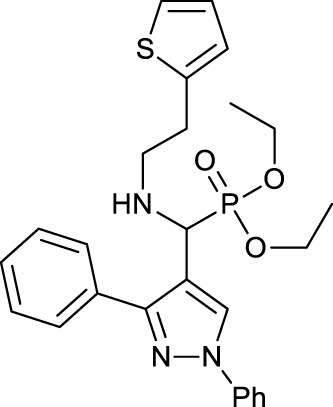	0.055 ± 0.143	47.46 ± 0.28	54.29 ± 0.126
**14b**	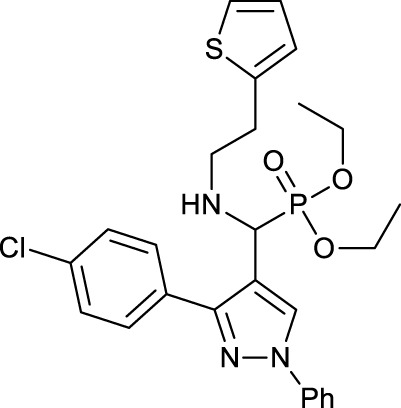	0.017 ± 0.02	46.48 ± 0.12	54.34 ± 0.064
Tacrine		0.210 ± 0.032	—	—
Galantamine		3.148 ± 0.139	—	—
Rivastigmine		2.632 ± 0.021	—	—
Ascorbic acid		—	45.64 ± 0.09	53.24 ± 0.064

**FIGURE 3:**
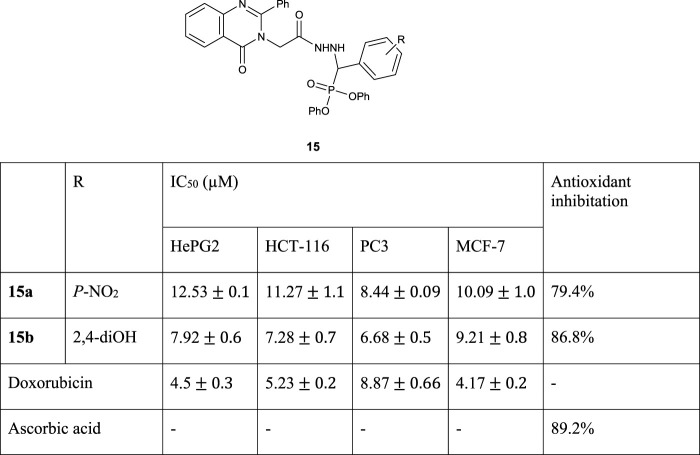
Structures of the compound **15** and its biological results.

Awad et al. (2018) reported the synthesis of compounds **15** through the one-pot Kabachnik–Fields reaction. They have examined their anticancer activities against HePG-2, MCF-7, HCT-116, and PC-3. The compounds **15a** and **15b** have good potential as anticancer agents. The results illustrate that compounds **15a** and **15b** had great antioxidant activities (Figure 3).

**FIGURE 4:**
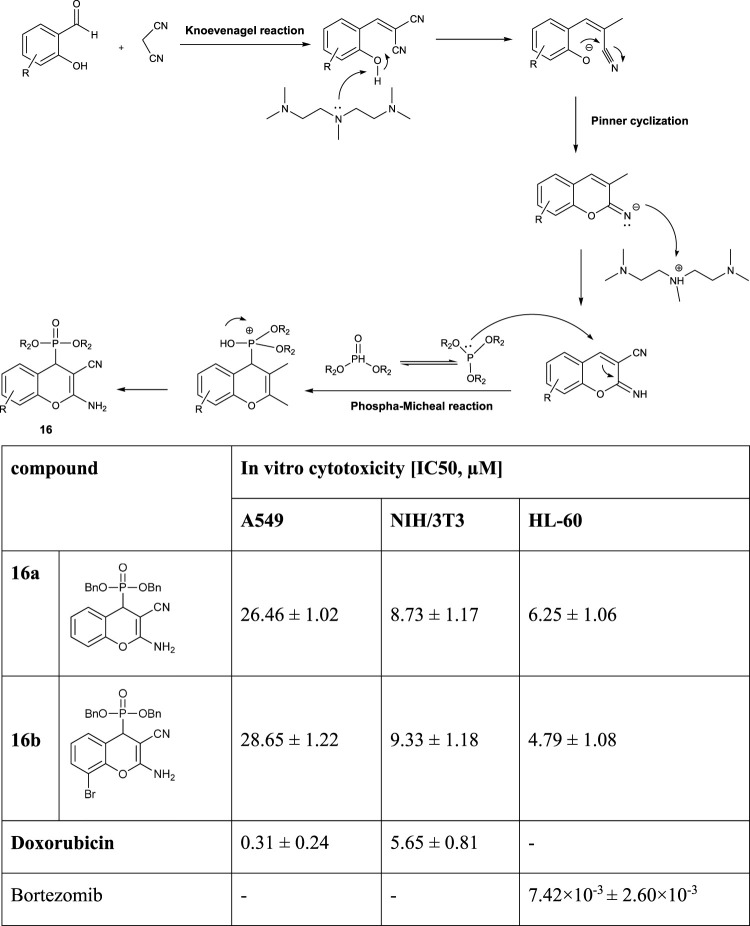
Structures of the compound **16** and its biological activities.

The compound **16** was synthesized by a three-component reaction of salicylaldehyde, malononitrile, and diethyl phosphite in the presence of 5 mol% of pentamethyldiethylenetriamine (PMDTA), under solvent-free conditions at 60°C. The *in vitro* cytotoxicity against human lung adenocarcinoma (A549), mouse fibroblasts (NIH/3T3) as a healthy cell line, and human promyelocytic leukemia (HL-60) has been studied. Among the synthesized aminophosphonates of **16**, compounds **16a** and **16b** exhibited the highest anticancer activities, but none of them worked better than doxorubicin and bortezomib (Figure 4) (Tajti et al., 2021).

The FDPS (farnesyl diphosphate synthase), an enzyme in the sterol biosynthetic pathway, catalyzes synthesis of farnesyl diphosphate from the reaction of geranyl diphosphate with isopentenyl diphosphate. The intracellular location of this enzyme is an important target for bisphophonate drugs (zoledronic acid and Alendronate). Four complexes [Pt(en)]_2_ZL, [Pt(en)]_2_IPrBP, [Pt(en)]_2_MIBP, and [Pt(en)]_2_EIBP dinuclear platinum was designed and synthesized based on imidazolyl-containing bisphosphonates **17–20** with high affinity for hydroxyapatite (Supplementary Scheme S9 in SI). However, these complexes showed a little inhibitory effect on bone cancer cells with less anticancer activity. They have better selectivity in inhibiting hepatocarcinoma cells than normal liver cells, especially complex **17** at high concentrations (100 μM) (Qiu et al., 2015).

Some of the bisphosphonates were used clinically, and their phosphorus esters were studied to evaluate how the structure of bisphosphonates affects bone attachment. Bisphosphonates like clodronate lost the ability to bind to hydroxyapatite by adding an ester group to them, unlike medronate. But hydroxy-bisphosphonates still retained their ability to bind even by binding to the two ester groups (Supplementary Scheme S10 in SI). Regarding the binding of bisphosphonates, the results were as follows (Supplementary Scheme S14): 1) a hydroxyl group in the geminal carbon participates in the bonding process and increases the ability of bisphosphonates to bind to the bone. 2) The ability of binding of bisphosphonates decreases with an increasing number of ester groups. 3) The location of ester groups has a significant effect on their ability to bind to bisphosphonates. (Puljula et al., 2015).

Hsiao and Wiemer (2018) investigated phospho antigens, including diphosphates, bisphosphonates, and precursors (Supplementary Scheme S11 in SI), for their ability to induce leukemia cells to stimulate the secretion of V*γ*9Vδ2 T-cell interferon-*γ*. Most of them showed their activity between 15 and 240 min. Potency (EC_50_ values) ranged between 8.4 nM and >100 μM. These findings showed better performance of prodrugs than other cases.

**FIGURE 5:**
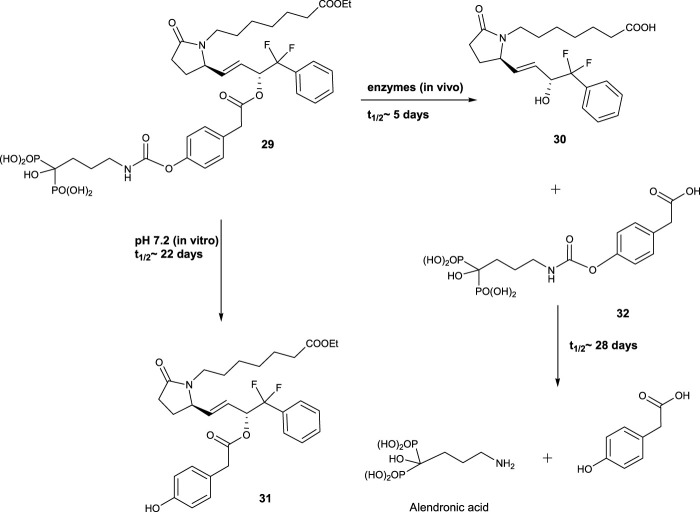
Potent EP4 receptor.

A potent EP4 receptor agonist was attached to the biologically inactive, bisphosphonate-based portion of the target bone. These single and doubly radiolabeled conjugates were made and showed to be stable in the blood, easily removed from the bloodstream, and effectively absorbed into the bone after *in vivo* dosing. It was found that doubly radiolabeled conjugate **29** splits widely between bone and liver, leaving the liver intact, and bone examination showed that the free EP4 agonist (compound **30**) was released from bone-bound **29** with a half-life of 7 days. The compound **29** binds rapidly and completely to powdered bone minerals or to various forms of calcium phosphate to form a stable matrix suitable for implantation. It can also be converted to powder or solid forms and sterilized without decomposing or releasing **29** (Figure 5) (Thévenin et al., 2021).

**FIGURE 6:**
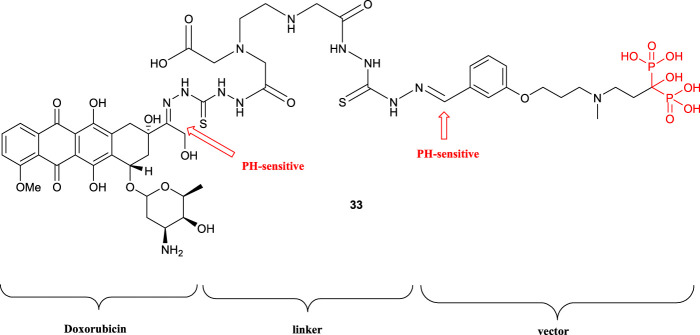
Structure of the compound **33**.

A new three building blocks including doxorubicin bound by the linker to the bone-targeted hydroxybisphosphonate vector through imine bonds (compound **33**) have been reported by David et al. (2019). Doxorubicin residue acts as an anticancer drug and the hydroxyl bisphosphonic acid group acts as a drug carrier to the target bone tissue. Due to the imine bond between doxorubicin and the vector to the linker, the doxorubicin drug was released in the target tissue with various acidic pH associated with the environment of the bone tumor. On the other hand, toxicity studies of compound **33** showed much less toxicity than doxorubicin. Furthermore, compound **33** had good effects on the osteosarcoma (Figure 6).

The covalent conjugation of the amino-bisphosphonate alendronate (ale) with the antimetabolite 5-fluoro 2′-deoxyuridine (5-FdU) is a new and effective drug to fight bone cancer. N^4^-(butyl-(4-hydroxy-4-phosphono) phosphate)-5-fluoro-2′deoxyuridine (5-FdU-alendronate, 5-FdU-ale) **34** has less toxicity than its two constituents *in vitro* and *in vivo* and is a promising candidate for the treatment of bone metastasis (Supplementary Scheme S12 in SI) (Schott et al., 2015).

Peng et al. (2017) showed that a novel zoledronic acid derivative **34** has antiproliferative and cytotoxic activity by inducing G1 cycle stopping and apoptosis and autophagy in the human colorectal cancer cell line HCT116. Induction of PTEN expression followed by inhibition of PI3K/Akt/mTOR is likely involved in these effects. These results provide a better understanding of the antitumor effects and underlying mechanisms of BPs in CRC treatment. Compound **35** had better antiproliferative effects on human CRC HCT116 cells than ZOL and may be used for CRC cancer therapy in the future (Supplementary Scheme S13 in SI).

**FIGURE 7:**
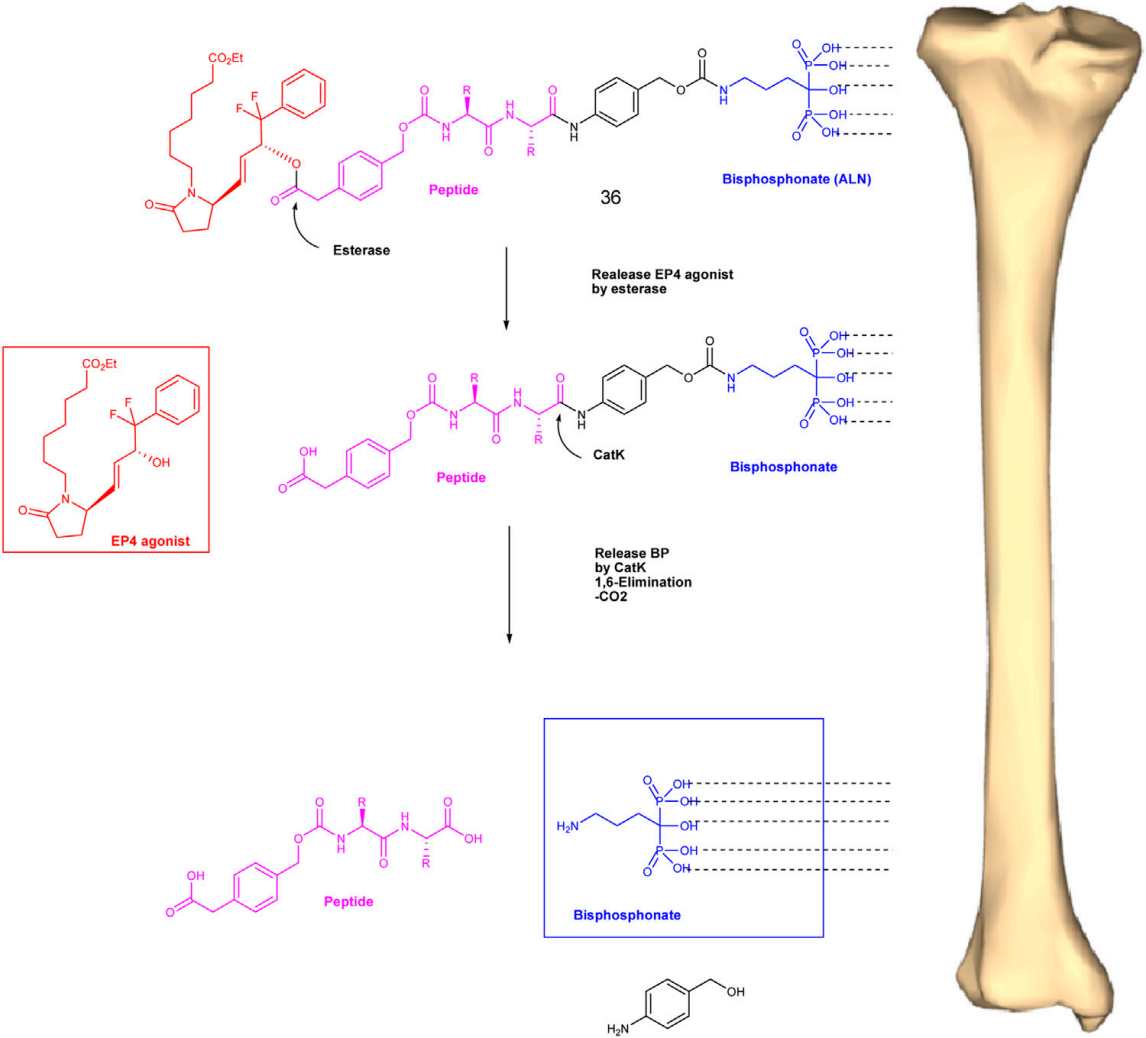
Conceptual differential enzymatic release of active EP4 agonist and bound bisphosphonate from the conjugate after binding to the bone surface.

A dual-action bone-targeting product **36** has been designed, synthesized, and evaluated for *in vitro* and *in vivo* metabolic stability by Xie et al. (2017). The compound was prepared from a combination of a highly potent anabolic selective agonist of the prostaglandin EP4 receptor and alendronic acid, a potent inhibitor of bone resorption optimally linked through a differentially hydrolyzable linker unit, N-4 carboxymethyl phenylmethyl oxycarbonyl-leucinyl-argininylpara aminolhen (Leu-Arg-PABA). It is designed to release the anabolic selective agonist activity of the prostaglandin EP4 receptor, and cathepsin K cleavage of the Leu-Arg-PABA element will release alendronic acid (Figure 7).

**FIGURE 8:**
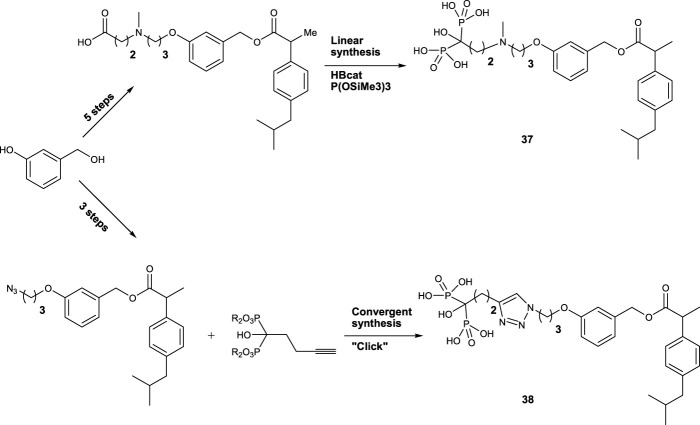
Synthesis of **37** and **38** as bone-targeting compounds.

Aoun et al. (2019) reported the synthesis and bone-targeting properties of a combination of terminal hydroxy-bisphosphonic function with a linear and convergent strategy (compounds **37** and **38**). Under neutral conditions using the Arbuzov reaction with tris (trimethylsilyl) phosphite and a carboxylic acid precursor activated *in situ* with catecholborane, free hydroxy-bisphosphonic was introduced in a linear approach (Figure 8).

By masking the negative charge of the P-C-P structure in bisphosphonates (BP) with pivoxil esters, their capacity to inhibit tumor cell growth can be increased (Supplementary Scheme S4 in SI, compounds **39–45**). BP pivoxil esters **39–45** are the most active structures for inhibiting the growth of hematopoietic cells with an IC_50_ value between 20 and 200 nM. In comparison, this value for zoledronic acid (Zol) is generally more than 20,000 nm. The compound tetrakispivaloyloxymethyl 2-(thiazole-2-ylamino)ethylidene-1,1-bisphosphonate (**45**) prevents the *in vitro* growth of tumor cells, especially hematopoietic cells at nm concentrations. Clinical studies have shown that it reduces the growth of human bladder cancer cells in a mouse model (Matsumoto et al., 2016).

Massarenti et al. (2017) reported the synthesis of novel aminobisphosphonates **46–48** as good candidates for antiresorption bone drugs (Supplementary Scheme S15 in SI).

### Enzyme inhibitors

Isoprenoid biosynthetic pathway (IBP) inhibitors include statins and nitrogenous bisphosphonates, which are used to treat bone disease and hypercholesterolemia. Reilly et al. (2015) reported the synthesis of the compound of disodium [(6z,11e,15e)-9-[bis(sodiooxy)phosphoryl]-17-hydroxy-2,6,12,16-tetramethylheptadeca-2,6,11,15-tetraen-9-yl] phosphonate **49** that selectively targets geranylgeranyl diphosphate synthase. Reduction of geranylgeranylation by the compound **49** inhibitor significantly reduces adrenal gland tumor metastasis *in vivo* (Supplementary Scheme S16 in SI).

A major therapeutic target for multiple myeloma is the geranylgeranyl diphosphate synthase enzyme. Inhibition of this enzyme disrupts protein geranylgeranylation, which disrupts intracellular protein trafficking. Some isoprenoid triazole bisphosphonates are potent and selective inhibitors of GGDPS. Bhuiyan et al. (2019) reported the synthesis and biological activities of some new analogs of triazole bisphosphonates to study their cellular and enzymatic activity. The compound **52** disrupts GGDPS with a minimal amount of inhibitory activity (Supplementary Scheme S17 in SI).

Malwal et al. (2019) reported the synthesis and biological activities of some novel bisphosphonates. After screening a library of inhibitors for biological activity against the long-chain prenyltransferase octaprenyl diphosphate synthase (OPPS), compound **51** showed IC_50_ values of ∼100 nM against heptaprenyl diphosphate synthase and 200 nM against a farnesyl diphosphate synthase (Supplementary Scheme S18 in SI).

Lacbay et al. (2018) reported the synthesis of a new category of thienopyrimidine-based bisphosphonate (ThP-BP) inhibitors **52–58** of the human geranylgeranyl pyrophosphate synthase (hGGPPS) that block protein prenylation in multiple myeloma (MM) cells leading to cellular apoptosis. These inhibitors are also effective in preventing the proliferation of other types of cancer cells. Administering a dose of ThP-BP inhibitor to a MM mouse model confirmed *in vivo* downregulation of Rap1A geranylgeranylation and reduction of monoclonal immunoglobulins (M-protein, a biomarker of disease burden) in the serum. According to the results, hGGPPS is a valuable therapeutic target in oncology and specifically for treating multiple myeloma (Supplementary Scheme S19 in SI).

### Inseticides, Antibacterials, and Antivirals

The insecticide *O,O′*-dimethyl (2,2,2-trichloro-1-hydroxyethyl) phosphonate **59**, commonly known as trichlorfon or metrifonate, is a well-known hydroxyphosphonate discovered in 1950. The compound **59** is the precursor for the synthesis of 2,2-dichlorovinyl dimethyl phosphate (DDVP) **60** that acts on flies, ticks, fleas, and cockroaches by inhibiting acetylcholinesterase enzyme. Rádai et al. (2018a) reported the synthesis and herbicidal activity of a new family of hydroxyphosphonates containing pyrimidine and quinoline scaffolds (**61** and **62**). Quinoline-based hydroxyphosphonate **62** was found to be an efficient antibiotic agent against Gram-positive (*Staphylococci* and *Bacillus megaterium-I*) as well as Gram-negative (E. coli, *Salmonella typhi*, and *Proteus vulgaris*) bacteria. The compound **63** showed a good antibacterial and antifungal activity against Gram-positive *Bacillus* subtilis and Gram-negative *E. coli* as well. A family of hydroxyphosphonates **64** containing the hydroxy group in the aromatic ring was reported as potential anticancer agents (Supplementary Scheme S20 in SI).

Zhang et al. (2019) reported the synthesis and herbicidal activities of some new structures of hydroxyphosphonate **65**. Studies showed that most of the prepared compounds with different substitutions have good herbicidal activity against Amaranthus retroflexus. Among them, the compounds (1-(5,5-dimethyl-2-oxido-1,3,2-dioxaphosphinan-2-yl)propyl-2-((4,6-dimethoxypyrimidin-2-yl)oxy)benzoate) **65a** and ((5,5-dimethyl-2-oxido-1,3,2-dioxaphosphinan-2-yl) (phenyl)methyl-2-((4,6-dimethoxypyrimidin-2-yl)oxy)benzoate) **65b** showed much higher post-emergency herbicide activity against monocotyledonous weeds (Supplementary Scheme S21 in SI).

**FIGURE 9:**
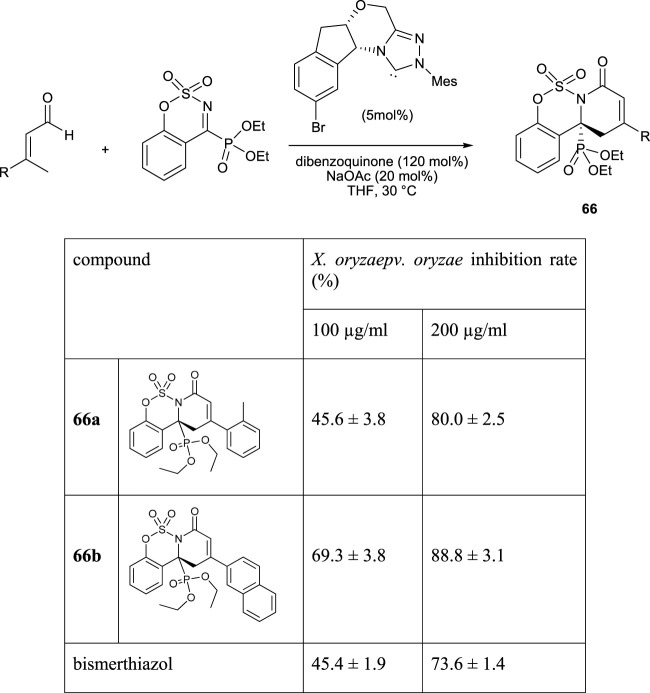
Structure of the compound **66**.

Sun et al. (2018) reported the synthesis of novel aminophosphonates *via* the aza-formal [4 + 2] cycloaddition reaction between the *β*-methyl-*α*,*β*-unsaturated aldehyde and the cyclic ketiminophosphonate in the presence of 5 mol% of NHC (N-heterocyclic carbene) as the chiral catalyst and 120 mol% of dibenzoquinone as the external oxidant. The antibacterial properties of synthesized products were assayed against *X. oryzae pv. oryzae*. This bacterium causes a serious disease named bacterial blight (BB) in rice plants. Among the synthesized aminophosphonates, compounds **66a** and **66b** had the most antibacterial activities (Figure 9).

Four compounds of aminophosphonates synthesized by the Kabachnik–Fields reaction in the presence of magnesium perchlorate as Lewis acid. The compounds were tested for their herbicidal and insecticidal activities. The compound **67** showed herbicidal activities against *Arabidopsis thaliana* and *Poa annua* the *in vitro* and against *Amaranthus retroflexus*, *Stellaria media*, *Lolium perenne*, and *Digitaria sanguinalis* the *in vivo* method. The results showed that some compounds such as **67** (Ar = Ph, 4-MeOC_6_H_4_-, 4-BrC_6_H_4_-, 2-ClC_6_H_4_-, 4-FC_6_H_4_-, and 2-BrC_6_H_4_-) displayed 100% inhibition against *Amaranthus retroflexus* at the dose of 1,000 g/ha in postemergence treatment. Compounds **67–70** did not exhibit any herbicidal activities. Some derivatives of the compound **70** displayed selective insecticidal activities against *Aphis species* or *Plutella xylostella* (Supplementary Scheme S22 in SI) (Chen et al., 2015).

**FIGURE 10:**
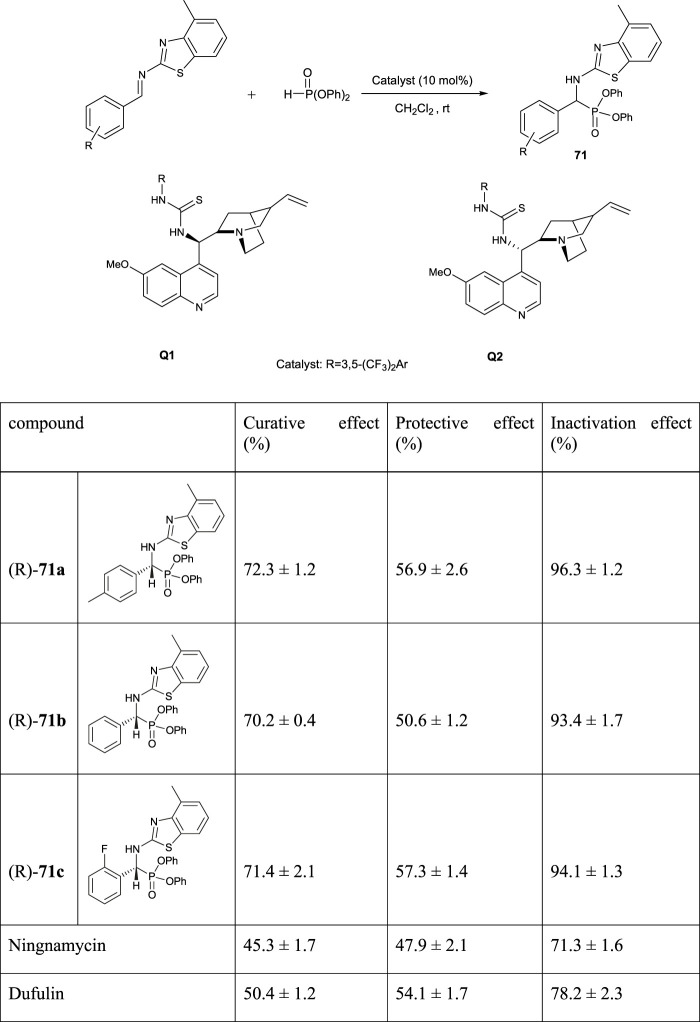
Synthesis and biological activities data of compounds **71**.

Asymmetric synthesis of dufulin-based aminophosphonates **71** was carried out using chiral thiourea organocatalysts (Q1 and Q2) with excellent enantioselectivity. The (R)-enantiomeric formed Q1, used as a catalyst, and the (S)-enantiomeric formed Q2. Antiviral activities of all prepared aminophosphonates were studied against cucumber mosaic virus (CMV). The results showed that (R)-enantiomers exhibited more antivirus activities than (S)-enantiomers. The outcomes of biological activity examinations illustrate that most of the synthesized compounds had antiviral potential. The compounds (R)-**71a**, (R)-**71b**, and (R)-**71c** even act better than dufulin and ningnanmycin (Figure 10) (Zhang et al., 2016).

*N*-Pyridylpyrazole derivatives of aminophosphonates **72** and **73** were synthesized and examined for their insecticidal and fungicidal activities. The results show that synthesized aminophosphonates displayed weak insecticidal activities toward *Mythimna separata* (Walker). Some of the compounds exhibited apparent fungicidal activity against six plant fungi such as *Fusarium oxysporum*, *Cercospora arachidicola*, and *Physalospora piricola* (Supplementary Scheme S23 in SI) (Wang et al., 2019).

The herbicidal activities of C-substituted derivatives of glyphosate **74** compared with pure glyphosate have been reported by Rogacz et al. (2020). All the tested aminophosphonic derivations were found to be safe for cultivated oat plants. Among the synthesized glyphosate derivatives, compound **74a** displayed the highest herbicidal activity against gallant soldier and common sorrel. It has herbicidal activities even more than pure glyphosate (Supplementary Scheme S24 in SI).

### Applications in Imaging

1-Hydroxy-2-(3-pyridyl)ethylidene bisphosphonic acid monosodium was synthesized with 71% yield and labeled with technetium-99m. The ^99m^Tc complex had a radiochemical purity of 99.2 ± 0.6% and was stable up to 6 h. The bio-distribution study showed a high absorption and long shelf life of the complex **75** in bone from 15 min (29 ± 2.5% ID/organ) to 4 h (35.1 ± 3.2 ID/organ) after injection. This provided a good tracker for bone imaging (Supplementary Scheme S25 in SI) (Motaleb et al., 2016).

**FIGURE 11:**
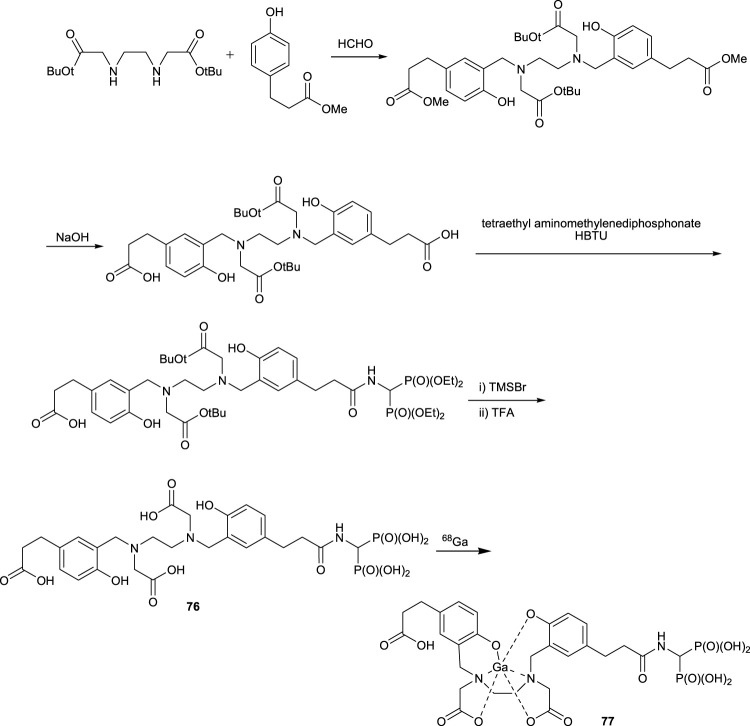
Synthesis of ligand **76** and complex **77**.

Positron emission tomography (PET) imaging with bisphosphonate-labeled ^68^Ga is a powerful tool for cancer diagnosis and monitoring treatment aimed at bone metastasis. *N,N*′-bis [2-Hydroxy-5-(carboxyethyl)benzyl]ethylenediamine-*N,N*′-diacetic acid ligand **76**, which contains a bisphosphonate group with a ^68^Ga label, was used as a valuable tool in bone imaging. Biodistribution and autoradiography studies of this combination showed that the tracer is taken up almost exclusively by the skeletal system with the least amount of accumulation of activity in other organs. This imaging agent can be used as a clinical tool to diagnose bone disorders and bone metastases in patients (Figure 11, complex **77**) (Zha et al., 2020).

Calcium minerals such as hydroxyapatite can be detected *in vivo* using nuclear imaging agents. Keeling et al. (2020) reported synthesis and application of novel hydroxyl bisphosphonic acid **78** in imaging. Radiolabeling of the ligand with ^68^Ga gave a high radiochemical yield and purity (>95%). The experiments results showed that all ^68^Ga-BPs have a high affinity for a broad range of calcium minerals implicated in vascular calcification disease. Furthermore, ^68^Ga-ligand showed high potential for clinical translation as a cyclotron-independent calcium mineral PET radiotracer (Supplementary Scheme S26 in SI).

### Other Miscellaneous Applications

**FIGURE 12:**
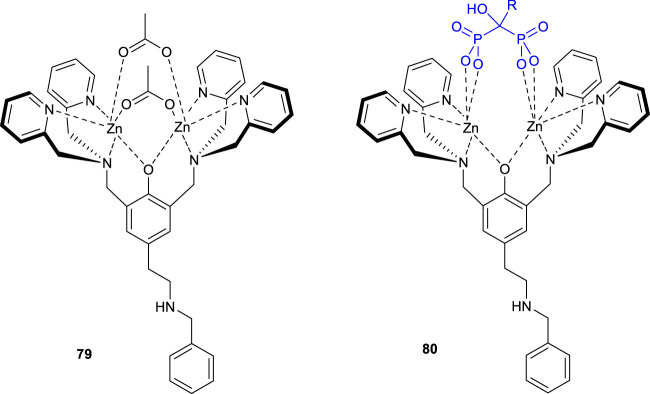
Structure of complexes **79** and **80**.

The di-zinc complex **79** binds to bisphosphonates with high affinity and good selectivity. The synthesized di-zinc(II) complex showed high affinity for bisphosphonates such as alendronate and etidronate. The prepared complex **80** was studied to treat a number of skeletal disorders and possessed anticancer properties. A modification with gold nanoparticles was used as a drug-loaded receptor with highly potential drug-delivery application. (Figure 12) (Torres-Huerta et al., 2020).

**FIGURE 13:**
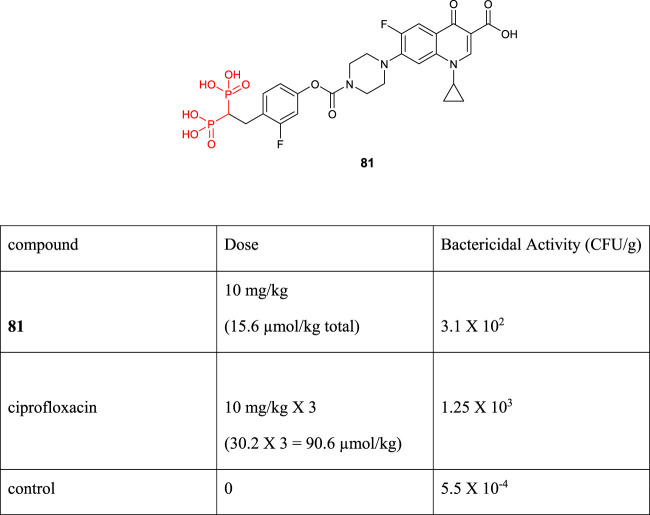
Structure of compound **81** and its biological activities.

The development of appropriate drug delivery to tissues is one of the most important research areas in recent decades. Osteomyelitis pathogens are biofilms attached to bone tissue that disrupts antibiotic delivery and is now known as the bone marrow problem. Sedghizadeh et al. (2017) reported the synthesis and drug-delivery properties of novel bisphosphonate-ciprofloxacin **81**. The “target and release” chemical strategy was used, and the results showed a significant therapeutic index against ciprofloxacin for the treatment of osteomyelitis *in vivo*. The stable release of the primary antibiotic occurred over time and increased the antibacterial effect of the leading drug in the target bone tissue (Figure 13).

Benyettou et al. (2015) reported a simple method for synthesis of a pH-sensitive drug-delivery system for doxorubicin (compound **82**). The drug was ligated to a novel silver nanocomposite, and the results of the doxorubicin release studies showed that the process occurred in late endosomes/lysosomes and became evenly distributed throughout the cytosol (Supplementary Scheme S27 in SI).

More than 65% of the elderly people have hearing loss problems. One of the important drugs for hearing problems is dihydroxyflavone (DHF). This drug is selective and protects the neuron from apoptosis due to the potent agonist of tropomyosin receptor kinase B (TrkB). Delivery of DHF to the inner ear is the main challenge. Kempfle et al. (2018) reported the synthesis of new bisphosphonate−dihydroxyflavone **83** for a novel approach to the targeted delivery of drugs to treat sensorineural hearing loss. Bisphosphonates residue of the compound **83** provides an exciting compound for targeted delivery due to its affinity for bone minerals, including cochlear bone. The results showed that the compound **83** is a convenient targeted delivery of dihydroxyflavone to treat sensorineural hearing loss (Supplementary Scheme S28 in SI).

**FIGURE 14:**
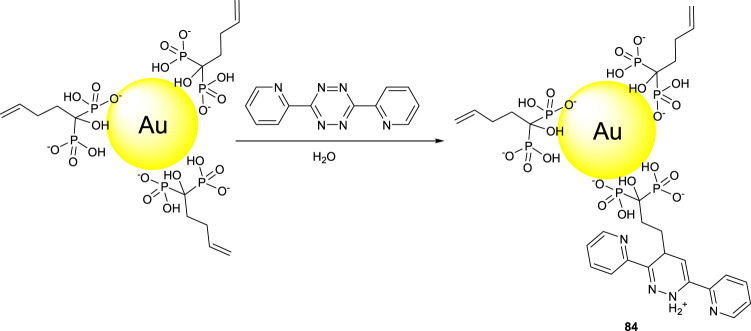
Synthesis of NIR probe **84**.

Aufaure et al. (2016) reported a novel NIR probe of Au nanoparticle-coated hydroxyl bisphohosphonate (compound **84**). 1-Hydroxy-1,1-methylenebisphosphonate bearing an alkene functionality was used for the preparation of gold nanoparticles bearing an alkene functionality for the synthesis of compound **84** *via* a tetrazine click chemistry (Figure 14).

## Conclusion

In summary, hydroxyl- and amino-phosphonates and -bisphosphonates are being developed due to their wide range of biological applications. Among them, hydroxybisphosphonates are gaining significant interest in pharmaceuticals due to their bone resorption inhibitors. These compounds are an important family of drugs from the clinical points in patients with metabolic bone disease. Aminobisphosphonates' properties are similar to hydroxybisphosphonates and therefore used as strong inhibitors of bone resorption for the treatment of Paget’s disease and osteoporosis. Hydroxy- and amino-phosphonates are being studied for enzyme inhibitors, anticancer properties, herbicides, and antibacterial properties. Different substitutions on the carbon atom connected to phosphorus have led to the synthesis of many phosphonates and bisphosphonates that have different physical, chemical, biological, therapeutic, and toxicological characteristics.
