# Supplementary material for: Hydroxy- and Amino-Phosphonates and -Bisphosphonates: Synthetic Methods and Their Biological Applications
Source: Front Chem. 2022 Jun 1;10:890696. doi: 10.3389/fchem.2022.890696 (PMC9200139; doi:10.3389/fchem.2022.890696)
Supplement: Supplementary file 1 [file DataSheet1.docx]

Hydroxy-and Amino-Phosphonates and Bisphosphonates: Synthetic Methods and their Biological Applications

Babak Kaboudin^1*^, Payam Daliri^1^, Samaneh Faghih^1^, and Hesam Esfandiari^1^

^1^Department of Chemistry, Institute for Advanced Studies in Basic Sciences, Gava Zang, Zanjan 45137-66731, Iran

*** Correspondence:**Babak Kaboudin

[kaboudin@iasbs.ac.ir](mailto:kaboudin@iasbs.ac.ir) and [kaboudin@gmail.com](mailto:kaboudin@gmail.com)

**Scheme 1**: The Pudovik method for the synthesis of hydroxyphosphonates **1**

**Scheme 2.** The Kabachnik-Fields synthesis of 1-aminophosphonates

**Scheme 3**. Synthesis of hydroxybisphosphonates

**Scheme 4**. Synthesis of aminobisphosphonates

**Scheme 5.** [3R,4R]-4-guanin-9-yl-3-((S)-2-hydroxy-2-phosphonoethyl)oxy-1-N-(phosphonopropionyl)pyrrolidine (**5**)

**Scheme 6**. Structure of the compound **6**

**Scheme 7.** Synthesis of the compounds **8-11**

**Scheme 8.** Synthesis of the compound **12**

**Scheme 9.** Pt complexes of bisphosphonates **17-20**

**Scheme 10.** Reactivity order of hydroxyl bisphosphonates **21-24** to bind to hydroxyapatite

**Scheme 11.** Phosphoantigen prodrugs

**Scheme 12**. Structure formula of 5-FdU-ale **34**

**Scheme 13.** Structure of compound **35**

**Scheme 14.** Structures of pivoxil esters **39-45**

**Scheme 15**. Novel candidates for anti-resorption bone drugs

**Scheme 17**. Structure of the compound **49**

**Scheme 17**. Structure of the compound **50**

**Scheme 18**. Structure of the compound **51**

**Scheme 19**. Structure of the compound **52-58** of thienopyrimidine-based bisphosphonate (ThP-BP) inhibitors

**Scheme 20**. Structure of the compound **59-64**

**Scheme 21**. Synthesis of the compound **65**

**Scheme 22**. Structure of the compounds **67-70**

**Scheme 23**. Synthesis of the compounds **72** and **73**

**Scheme 24**. Structures of the compound **74**

**Scheme 25**. ^99m^Tc-BPs structure of complex **75**

**Scheme 26**. Structure of ligand **78** for bone imaging

**Scheme 27**. Structure of the compound nanocomposite **82**

**Scheme 28**. Structure of the novel DHF carrier **83**
